# Elevated CO_2_ and Increased N Intensify Competition between Two Invasive Annual Plants in China

**DOI:** 10.3390/life12101669

**Published:** 2022-10-21

**Authors:** Caiyun Zhao, Xiangjian Zhao, Junsheng Li

**Affiliations:** 1 State Key Laboratory of Environmental Criteria and Risk Assessment, Chinese Research Academy of Environmental Sciences, Beijing 100012, China; 2 China National Accreditation Service for Conformity Assessment, Beijing 100062, China; 3 Command Center for Comprehensive Survey of Natural Resources, China Geological Survey Bureau, Beijing 100055, China

**Keywords:** elevated CO_2_, increased N, common ragweed, redroot pigweed, invasional interference, interspecific competition

## Abstract

As multiple invaders often co-occur, understanding the interactions between different invasive species is important. Previous studies have reported on invasional meltdown and neutral and interference relationships between invasive species. However, interspecific interactions may vary with environmental change owing to the different responses of interacting invaders. To better understand the interaction of notorious invasive alien plants under CO_2_ enrichment and N deposition, the growth characteristics of common ragweed (*Ambrosia artemisiifolia*) and redroot pigweed (*Amaranthus retroflexus*) were studied when they were planted in monoculture (4Rag and 4Pig) or mixture (1Rag:3Pig, 2Rag:2Pig, 3Rag:1Pig) under four environmental treatments: elevated CO_2_, increased N, elevated CO_2_ + increased N and a control. Increased N positively affected almost all the traits (basal stem diameter, height, shoot biomass, root biomass and total biomass) of common ragweed, except for branch number and root-shoot ratio. But increased N only promoted redroot pigweed’s height and basal stem diameter. interspecific competition promoted basal stem diameter and number of branches but decreased root biomass of common ragweed, and the basal stem diameter was significantly higher in 1Rag:3Pig and 2Rag:2Pig compared to the other two treatments. interspecific competition inhibited almost all the characteristics of redroot pigweed. The interaction between elevated CO_2_ and increased N also increased the biomass characteristics (shoot biomass, root biomass and total biomass) of common ragweed. However, elevated CO_2_ inhibited the root biomass of redroot pigweed. The results indicated that common ragweed was a superior competitor under conditions of elevated CO_2_ and increased N. Moreover, environmental change might strengthen the super-invasive plant common ragweed’s competitive ability.

## 1. Introduction

Invasive plants often show high adaptability and phenotypic plasticity, which allow them to thrive under altered environmental conditions [1,2]. Numerous case studies have demonstrated that invasive plants benefit from increasing N deposition and elevated CO_2_ [3,4,5,6]. Elevated CO_2_ can facilitate plant invasion by increasing plant photosynthesis, growth rates, efficient resource use, productivity, and seed production [7,8,9,10,11,12]. N deposition can facilitate plant invasion by increasing N availability, plant growth, and competitive ability [13,14,15,16]. These factors often change simultaneously. However, in most studies, these two factors are studied independently.

Understanding the interactive effect of elevated CO_2_ and N deposition on invasive alien plants is important. Recent research demonstrated that elevated CO_2_ and N deposition synergistically increased the performance (such as biomass and size, survival and reproduction, and photosynthetic rate) of invasive [4,17,18] or naturalized alien plants [6] than native. Previous studies tested the effect of elevated CO_2_ and N deposition on alien plants compared with native plants, but few studies have tested the effect among various invasive plants. 

As biological invasions increase in frequency, most habitats are invaded by multiple invasive plants, and interactions between invasive species have attracted attention [19,20,21]. Thus far, three types of interactions have been described: (1) Establishment and impact of one alien invasive species can be facilitated by another invasive species, which is described as invasional meltdown [22]; (2) neutral interactions have also been described [23]; (3) invasion by one species can be negatively impacted by the presence of another invader, termed invasional interference [24,25,26]. Many authors have noted that a decline in one nonnative species results in a rapid increase in another, which indicates that competition among invasive plants may be common [21,27]. In some cases, these negative relationships may result in an invasive species replacing another invasive species, termed “over-invasion” [20]. For example, the replacement of *Spartina anglica* by *S. alterniflora* was found in China [28]. Interactions between invasive plants under elevated CO_2_ and N deposition remain unclear.

Common ragweed and redroot pigweed are two notorious invasive species both native to North America. They are both included on the list published by the Ministry of Ecology and Environment of the People’s Republic of China [29]. Common ragweed, an annual weed in crop fields, usually forms dense mono-specific stands and produces a considerable amount of pollen [30], and this weed is one of the most problematic aero-allergens [31]. It was introduced into China in the 1930s and, since then, it has spread across twenty provinces [32]. Redroot pigweed occurs in various habitats, including agricultural and ruderal habitats [25,33]. It has a negative impact on ecosystems and native species [34,35] and is regarded as the third most notorious weed in the world [25,36]. Redroot pigweed, introduced into China around 1905, has expanded its distribution in large areas [37]. These two invasive species often co-occur in crops or other habitats [38,39]. 

In the present study, we tested competitive interactions between these two invasive alien plants in monoculture and mixture under four environmental treatments: elevated CO_2_, increased N, elevated CO_2_ and N, and a control. We aimed to answer the following questions: (1) do the growth characteristics of these two invasive alien plants respond to elevated CO_2,_ increased N, and replacement in the same way? (2) which invasive species is more competitive under elevated CO_2_, increased N deposition, and replacement? (3) how do elevated CO_2,_ N deposition, and replacement affect the reproduction of common ragweed?

## 2. Materials and Methods

### 2.1. Plant Materials

We collected seeds of common ragweed and redroot pigweed from Mentougou District in Beijing (100 km from the experiment site) in October 2013. The seeds were treated at low temperatures (−20 °C) for two months to break dormancy and then stored at room temperature in paper bags. The seeds of the two species were sown on 7 May 2014, at a depth of 2 cm in two field plots and then watered to field capacity once to stimulate germination. After three weeks, seedlings were transplanted into pots in the OTCs according to the experimental design.

### 2.2. Experimental Design

The experiment was conducted at the Chinese Research Academy of Environmental Sciences field laboratory, Shunyi District, Beijing, China (116.5875° E, 40.19° N), between 5 June and 8 October 2014.

We tested two CO_2_ concentrations in our experiment: ambient CO_2_ (375 ppm) concentrations and elevated CO_2_ (700 ppm) based on IPCC (Intergovernmental Panel on Climate Change) [40]. Four pairs of open-top chambers (OTCs: 2.2 m in height with an octagonal ground surface area of 6.25 m^2^) were used. During the experiment, pure CO_2_ was continuously ventilated into the OTCs of elevated CO_2_ treatment. Elevated CO_2_ concentrations were measured at 5-min intervals by monitoring sensors (Qs100). In the elevated CO_2_ treatment, the achieved level was 695.00 ± 15.67 (mean ± SD) ppm. Ambient CO_2_ and elevated CO_2_ were randomly assigned to each pair. According to the increase in N deposition rates over the coming decades in China [41,42], two N levels were conducted: ambient N (0 addition) and increased (N 0. 8 g/pot).

**Figure 1:**
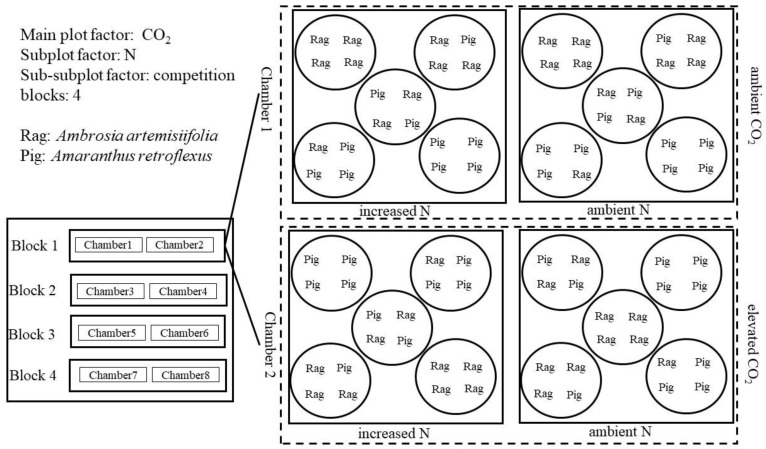
Experimental design in this study.

Four seedlings were planted in each pot under the replacement design: 4Rag:0Pig, 1Rag:3Pig, 2Rag:2Pig, 3Rag:1Pig, and 0Rag:4Pig (where Rag and Pig denote common ragweed and redroot pigweed, respectively). Two N levels, and five replacement levels within each N level, produced 10 pots in each chamber. Thus, 80 pots (32 cm in diameter and 38 cm in depth) were randomly arranged within 8 OTC in the following experimental design: 2 CO_2_ levels × 2 N levels × 5 replacement levels × 4 replicates (Figure 1). During the growing season, N was equally divided eight times and uniformly applied in the form of NH_4_NO_3_ solution, while the control pots were sprayed with the same volume of water.

To ensure homogeneity of the growing conditions, the pots were filled with a mixture of local soil (80%; collected at a depth of 3~15 cm from a weedy field neighboring the experiment base after excluding topsoil) and vermiculite (20%). Before transplanting, the following soil nutrient contents were measured: organic carbon, 8.4 g·kg^−1^; total N, 0.73 g·kg^−1^; ammonium N, 10.77 mg·kg^−1^; and nitrate N, 6.53 mg·kg^−1^. One week after transplanting, the elevated CO_2_ and N enrichment treatments were initiated. Plants were watered weekly as needed throughout the experiment. In each OTC, pots were rearranged randomly within the chamber every month to minimize location-specific effects.

### 2.3. Measurements and Calculations

Plant height, basal stem diameter, and branch number per plant of common ragweed and redroot pigweed were measured in October. Shoots of each species in every pot were harvested from above the soil surface and stored in archival paper bags. We turned over the pot, removed the soil of roots with running water, carefully separated the roots of two species in every pot, and then stored them in paper bags. The shoots and roots were oven-dried at 80 °C for 72 h. Shoot biomass and root biomass of each species in each pot were measured, and the total biomass of each species was calculated per pot.

The competitive ability of these two invasive plants was measured by relative yield. RY values > 1 indicate that one species does better when competing against the other species than when competing against itself [43]. RY values were calculated using the equation below [43,44].

$$RY_{i}=\frac{Y_{ij}}{p_{i}\times Y_{i}}$$

where *Y_ij_* is the yield of species *i* in the presence of species *j*, *p_i_* is the proportion at which species *i* is sown, and *Y_i_* is the yield of species *i* in monoculture under the same CO_2_ and N treatment as that for *Y_ij_*.

To analyze the impact of elevated CO_2_, increased N, and replacement on the reproduction of common ragweed, in each pot with common ragweed, seeds were collected by hand in October when the seeds were mature but had not yet begun to drop. The total seed number and the total seed weight were measured as seed yield.

### 2.4. Statistical Analysis

Split-plot ANOVA was employed to test the effects of CO_2_, N, and replacement on the plant performance and relative yield of common ragweed and redroot pigweed, with block as a random factor. When replacement level had a significant effect, significant differences between replacement levels were tested using the Tukey honesty significant difference post-hoc analyses (HSD) (*p* < 0.05). In all ANOVAs, data were log-transformed to conform to the assumptions of normality and homoscedasticity. All analyses were performed using IBM SPSS Statistics 19 (IBM, 2010, New York, NY, USA).

## 3. Results

### 3.1. Growth Characteristics of Two Invasive Alien Plants

**Figure 2:**
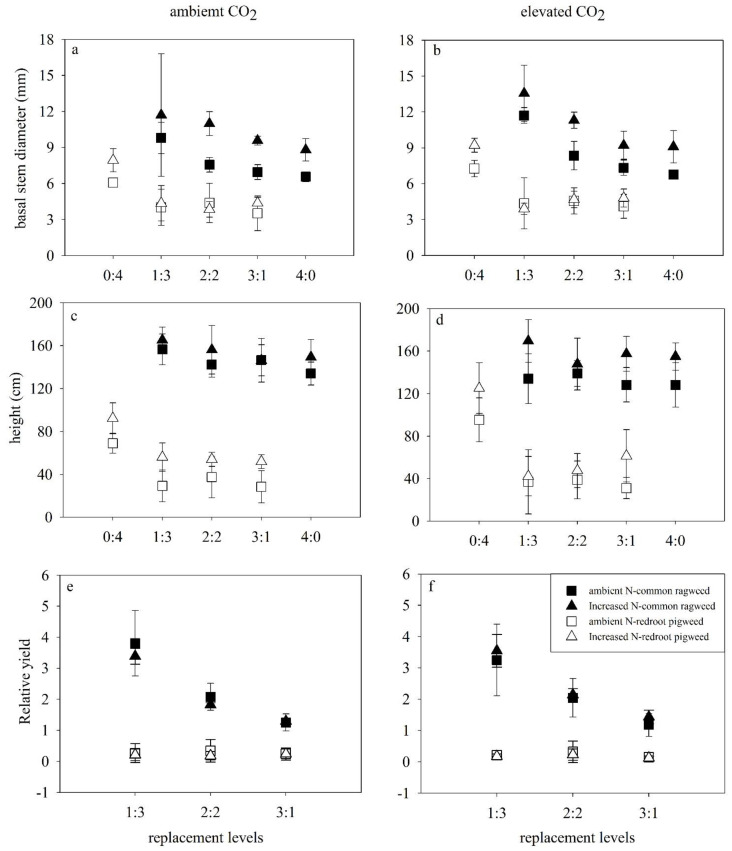
Effects of elevated CO_2_ (ambient CO_2_, left; elevated CO_2_, right), increased N and replacement levels on the growth characteristics of two invasive alien plants. Bars show mean ± SD (4). (**a**,**b**). Basal stem diameter. (**c**,**d**) Height. (**e**,**f**) RY. On the horizontal axis, 1:3 refers to one common ragweed plant and three redroot pigweed plants, 2:2 refers to two common ragweed plants and two redroot pigweed plants, 3:1 refers to three common ragweed plants and one redroot pigweed plant, and 0:4 and 4:0 refer to four redroot pigweed plants or four common ragweed plants, respectively.

Results revealed that height and basal stem diameter of common ragweed were significantly enhanced by increased N in both monoculture and mixtures under both CO_2_ concentrations (*p* < 0.001) (Figure 2a–d; Table 1). At the same time, increased N enhanced (*p* < 0.05) the height and basal stem diameter (*p* < 0.001) of redroot pigweed.

Species replacement (*p* < 0.001) also promoted the basal stem diameter of common ragweed, which was significantly larger in the mixture than in monoculture. The mean stem diameter of ragweed was the largest in the 1Rag:3Pig (*p* < 0.05, HSD test) (Figure 2a,b), and also significantly larger in the 2Rag:2Pig than in monoculture (*p* = 0.014, HSD test). Species replacement (*p* < 0.001) decreased the height and basal stem diameter of redroot pigweed (Figure 2a–d, Table 1). These two characteristics of redroot pigweed in monoculture were all significantly larger than all mixture treatments (*p* < 0.001, Figure 2a–d; HSD test). Species replacement increased the branch number of common ragweed (*p* < 0.001), but no branches were observed for redroot pigweed during the experiment.

The impacts of elevated CO_2_ on these two invasive plants were not found. A significant interaction of impacts on redroot pigweed was detected in increased N and competition (*p* = 0.041), elevated CO_2_ and species replacement (*p* = 0.019).

### 3.2. Biomass Characteristics of Two Invasive Alien Plants

**Figure 3:**
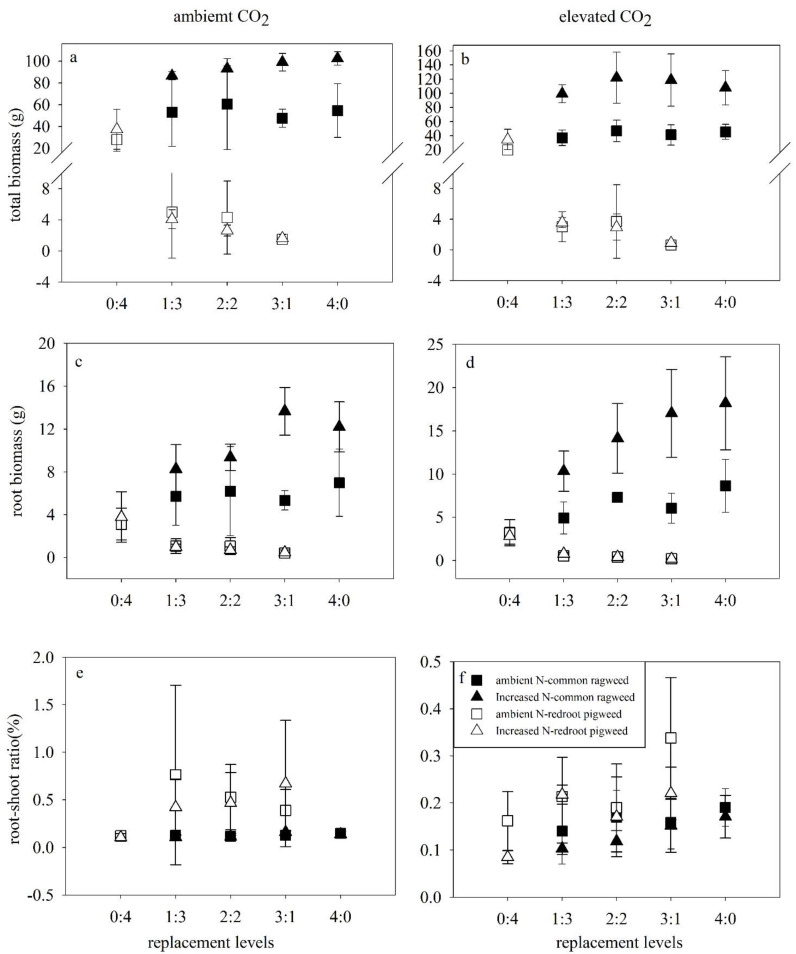
Effects of elevated CO_2_ (ambient CO_2_, left; elevated CO_2_, right), N addition and replacement levels on the biomass of two invasive alien plants. Bars show mean ± SD (4). (**a**,**b**). Total biomass. (**c**,**d**). Root biomass. (**e**,**f**). Root–shoot ratio. On the horizontal axis, 1:3 refers to one common ragweed plant and three redroot pigweed plants, 2:2 refers to two common ragweed plants and two redroot pigweed plants, 3:1 refers to three common ragweed plants and one redroot pigweed plant, and 0:4 and 4:0 refer to four redroot pigweed plants or four common ragweed plants, respectively.

Shoot biomass, root biomass, and total biomass of common ragweed were all significantly increased by N addition in both monoculture and mixtures under both CO_2_ concentrations (Figure 3a–d; Table 2). No impacts of N addition were found in biomass characters of redroot pigweed.

Root biomass and the root-shoot ratio of common ragweed were affected by species replacement. Interspecific and intraspecific competition all impacted root biomass and were higher in monoculture (*p* = 0.001, HSD test) and decreased in 3Rag:1Pig (*p* = 0.020, HSD test) than in 1Rag:3Pig mixture. The root-shoot ratio of common ragweed was significantly lower in the 3Rag:1Pig mixture than in monoculture (*p* = 0.011, HSD test). Shoot biomass, root biomass, and total biomass of redroot pigweed were all significantly suppressed in the mixture compared with monoculture (*p* < 0.001) (Figure 3a–d; Table 2), and these characteristics were all decreased in 3Rag:1Pig than in the other two mixture controls (*p* < 0.05, HSD test). The root-shoot ratio of redroot pigweed was significantly higher in mixtures than in monoculture (*p* < 0.05, HSD test) (Figure 3e, f).

Elevated CO_2_ inhibited the root biomass (*p* = 0.011) of redroot pigweed in mixtures (Figure 3c, d). An interaction between elevated CO_2_ and increased N promoted shoot biomass (*p* < 0.001), total biomass (*p* < 0.001), and root biomass (*p* = 0.03) of common ragweed.

### 3.3. Relative Yields of Two Invasive Alien Species

The relative yields of common ragweed were significantly higher than 1 (*p* < 0.001, *t*-test) irrespective of any treatments; while these values were significantly lower than 1 (*p* < 0.001, *t*-test) for redroot pigweed (Figure 2e,f). The highest relative yield (RY) values of common ragweed reached 4.72, and the mean values of relative yield were larger in 1Rag:3Pig compared to other replacement treatments (*p* < 0.001, HSD test), while these values in 2Rag:2Pig were higher than in 3Rag:1Pig (*p* = 0.001, HSD test). In contrast, the largest relative yield of redroot pigweed was only 0.87, and the mean values of relative yield were higher in 2Rag:2Pig compared to other replacement treatments; however, no significant difference was observed.

### 3.4. Reproductive Characteristics of Common Ragweed

**Figure 4:**
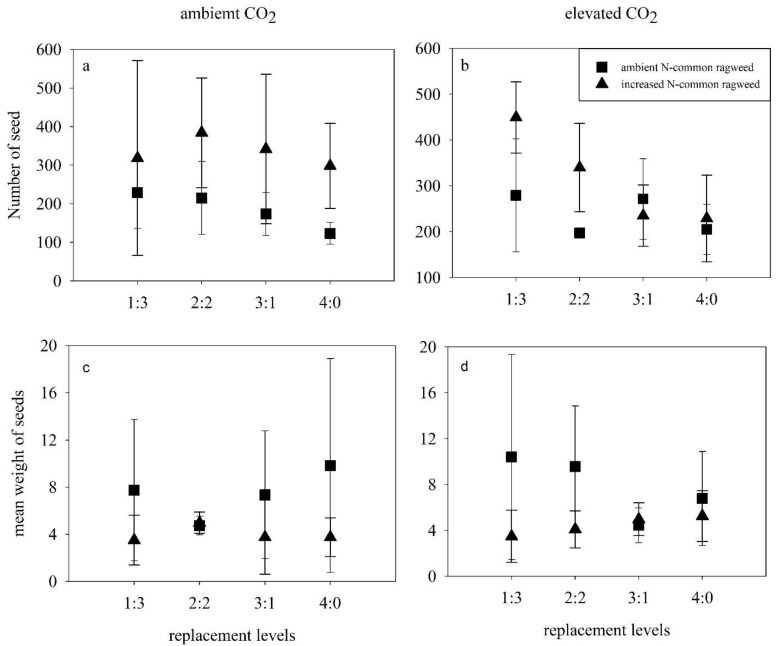
Effects of elevated CO_2_, N addition and replacement levels on the reproductive characteristics of common ragweed. Bars show mean ± SD (4). (**a**,**b**) The number of seeds (**c**,**d**) mean weight of seeds. On the horizontal axis, 1:3 refers to one common ragweed plant and three redroot pigweed plants, 2:2 refers to two common ragweed plants and two redroot pigweed plants, 3:1 refers to three common ragweed plants and one redroot pigweed plant, and 4:0 refers to four common ragweed plants.

Increased N enhanced the seed yield (*p* < 0.001; Table 3; Figure 4a,b) and decreased the seed mean weight (*p* = 0.001) of common ragweed (Figure 4c, d), while elevated CO_2_, competition, and interaction effects showed no significant difference on any of the abovementioned indices (Figure 4; Table 3).

## 4. Discussion

In the present study, we found common ragweed and redroot pigweed responded differently to elevated CO_2_, increased N, and species replacement. We revealed the interspecific competition between common ragweed and redroot pigweed under elevated CO_2_ and increased N.

Common ragweed showed an apparent competitive advantage over redroot pigweed under mixture treatments, where even one common ragweed plant could strongly inhibit redroot pigweed plants. Common ragweed and redroot pigweed may compete for limited resources. The tall statue of the common ragweed provided a decisive advantage in acquiring light [45]. Common ragweed was higher and more robust than redroot pigweed in mixture treatments (Figure 1 and Figure 2). The height disadvantage of redroot pigweed under competition results in a decrease in its light-capturing capacity [46]. The decline in quality and quantity of light influenced the phenology of pigweed [35,47], so the growth of redroot pigweed was inhibited under competition. We also found the height of redroot pigweed are all positively related to the other characteristics. By comparing the competition between redroot pigweed and common lamb’s quarters (*Chenopodium album*), it was also found that the species which shade the ground with a dense canopy will favorably compete [48]. In our study, we also found that branch numbers of common ragweed increased with competition, but no branches were observed in redroot pigweed. When two alien invasive plants live in the same habitat or require the same scarce resource [49,50], one species limiting the material or space of the other will result in competition.

Growth characteristics (except for branch number and root-shoot ratio) of common ragweed were enhanced by increased N in all treatments. Only the height and basal stem diameter of redroot pigweed increased in the mixtures under increased N (Figure 1 and Figure 2). Our results are consistent with the premise that responsiveness considerably differs between species under N addition [51]. We also found that increased N and elevated CO_2_ intensified the competitive advantage of common ragweed. For example, biomass characteristics of common ragweed were positively affected by the interaction of increased N and elevated CO_2_. Lack of an elevated CO_2_ direct effect on these two invasive alien plants might be explained by indirect effects of CO_2_ on N limitation, similar to Blumenthal et al. [52]. First, these two invasive alien plants all prefer high levels of nitrogen fertilizer. A previous study showed that the height and dry matter of common ragweed were increased by N addition in both greenhouse and field experiments [53]. Moreover, N addition stimulated the height of redroot pigweed [35] and shoot biomass and root biomass [51]. In the present study, the biomass of redroot pigweed did not increase under N addition in mixtures. Mainly because common ragweed can competitively pre-empt soil nitrogen from redroot pigweed. Secondly, the CO_2_ response of species might depend on local resource availability in mixed-species competition [4]. Most plants exhibit a positive growth response to elevated CO_2_ due to increased photosynthesis and/or efficient nutrient use when other factors (e.g., water and nutrients) are not limited [6,54]. Because most invasive plants are sensitive to N availability, the impacts of CO_2_ on invasive plants varied with N levels [52]. Redroot pigweed increased biomass allocation to the roots to diminish aboveground competitive disadvantage, where plants allocate more biomass to roots to acquire the most limiting resource [55,56,57]. But elevated CO_2_ inhibits this increase (Figure 2). A previous study on redroot pigweed revealed lower rates of photosynthesis and stomatal conductivity under elevated CO_2_ compared to ambient in water stress treatments [58,59]. We speculate that there is an apparent limit of available N for redroot pigweed growth under competition. Third, C3 plants are thought to take more advantage of CO_2_ enrichment than plants with a C4 [60,61]. In our study, common ragweed was a C3 plant and redroot was a C4 plant. Fourth, allelopathic effects also impacted interspecific competition. Bae et al. found that elevated CO_2_ may enhance the allelopathic potential of common ragweed [62].

The competition outcome varies with the performance [45] and resource availability of neighboring species [63]. For example, Italian ryegrass has a competitive advantage over common ragweed [64], while redroot pigweed shows higher competitiveness than *Phaseolus vulgaris* [65]. Competitiveness decreased with an increasing density of common ragweed. The highest competitiveness was under 1Rag:3Pig, where the relative yield of common ragweed increased to 350%. Results indicated interspecific and intraspecific generality, as detected in other experiments [4]. It is also possible that release from intraspecific competition allows common ragweed to grow higher and more robust. In contrast, their biomass decreased when redroot pigweed was released from the intraspecific competition (Figure 1 and Figure 2). Intra- or con-specific competition is always stronger than interspecific one [66,67]. Previous studies have shown that elevated CO_2_ and N deposition might favor performances of invasive plants relative to that of native species [3]. Our results revealed elevated CO_2_ and increased N prefer the super competitor when two notorious alien invasive plants grew together.

## 5. Conclusions

The purpose of this study was to understand the differences in growth characteristics of two alien invasive plants under elevated CO_2_, increased N, and species replacement. The results showed that the biomass of common ragweed was positively enhanced by increased N and species replacement, but redroot pigweed was negatively inhibited. The relative yield of these two notorious invasive plants revealed a competition interaction. In addition, our results revealed that common ragweed gained a more competitive advantage than redroot pigweed under increased N and elevated CO_2_. Our results indicated that common ragweed may be replaced redroot pigweed in heavily invaded regions. The competition or coexistence of redroot pigweed and common ragweed should be discussed under different invasion stages in the future, especially under environmental change. Moreover, understanding the interaction of invasive alien species will help us to manage multispecies invasions in the future.

## Figures and Tables

**Table 1:** Results of three-way ANOVA for the growth characters of the alien invasive plants common ragweed and redroot pigweed according to CO_2_, N and replacement levels: basal stem diameter, height, branch number.

Species	Factors		Basal Stem Diameter	Height	Branch Number
common ragweed	CO_2_	*F*	2.756	3.881	0.84
		*p*	0.104	0.055	0.364
		*df*	1	1	1
	N	*F*	32.843	17.834	0.077
		*p*	<0.001 **	<0.001 **	0.783
		*df*	1	1	1
	replacement	*F*	14.961	2.446	15.668
		*p*	<0.001 **	0.076	<0.001 **
		*df*	3	3	3
	CO_2_ × N	*F*	0.102	3.65	2.025
		*p*	0.751	0.062	0.161
		*df*	1	1	1
	N × replacement	*F*	1.163	0.398	2.198
		*p*	0.334	0.755	0.101
		*df*	3	3	3
	CO_2_ × replacement	*F*	1.093	0.222	0.905
		*p*	0.361	0.881	0.446
		*df*	3	3	3
	CO_2_ × N × replacement	*F*	0.128	0.944	1.094
		*p*	0.943	0.427	0.361
		*df*	3	3	3
redroot pigweed	CO_2_	*F*	0	3.405	-
		*p*	0.994	0.071	-
		*df*	1	1	
	N	*F*	4.599	26.336	-
		*p*	0.037 *	<0.001 **	-
		*df*	1	1	
	replacement	*F*	36.172	43.233	-
		*p*	<0.001 **	<0.001 **	-
		*df*	3	3	
	CO_2_ × N	*F*	0.009	0.249	-
		*p*	0.926	0.62	-
		*df*	1	1	
	N × replacement	*F*	2.971	0.834	-
		*p*	0.041 *	0.482	-
		*df*	3	3	
	CO_2_ × replacement	*F*	0.896	3.64	-
		*p*	0.45	0.019 *	-
		*df*	3	3	
	CO_2_ × N × replacement	*F*	0.289	0.712	-
		*p*	0.833	0.55	-
		*df*	3	3	

Df represents degrees of freedom, * represents *p* < 0.05, ** represents *p* < 0.01

**Table 2:** Results of three-way ANOVA for the biomass characters of the alien invasive plants common ragweed and redroot pigweed according to CO_2_, N and replacement levels: shoot biomass, root biomass, total biomass, and root–shoot ratio.

Species	Factors		Shoot Biomass	Root Biomass	Total Biomass	Root-Shoot Ratio
common ragweed	CO_2_	*F*	2.141	0.855	2.107	0.042
		*p*	0.15	0.36	0.153	0.839
		*df*	1	1	1	1
	N	*F*	140.242	73.943	142.018	2.843
		*p*	<0.001 **	<0.001 **	<0.001 **	0.099
		*df*	1	1	1	1
	replacement	*F*	1.085	5.75	1.398	5.158
		*p*	0.365	0.002 **	0.255	0.004 **
		*df*	3	3	3	3
	CO_2_ × N	*F*	13.565	4.989	13.245	2.609
		*p*	0.001 **	0.030 *	0.001 **	0.113
		*df*	1	1	1	1
	N × replacement	*F*	0.399	2.804	0.651	1.275
		*p*	0.754	0.05	0.587	0.294
		*df*	3	3	3	3
	CO_2_ × replacement	*F*	0.361	0.808	0.346	0.806
		*p*	0.781	0.496	0.792	0.497
		*df*	3	3	3	3
	CO_2_ × N × replacement	*F*	0.354	0.064	0.271	0.294
		*p*	0.786	0.979	0.846	0.83
		*df*	3	3	3	3
redroot pigweed	CO_2_	*F*	1.92	5.08	2.63	0.037
		*p*	0.173	0.029 *	0.112	0.849
		*df*	1	1	1	1
	N	*F*	1.614	0.013	1.637	0.383
		*p*	0.21	0.909	0.207	0.539
		*df*	1	1	1	1
	replacement	*F*	100.968	57.074	110.475	4.281
		*p*	<0.001 **	<0.001 **	<0.001 **	0.010 **
		*df*	3	3	3	3
	CO_2_ × N	*F*	0.588	0.054	0.636	0.088
		*p*	0.447	0.818	0.429	0.768
		*df*	1	1	1	1
	N × replacement	*F*	0.512	0.182	0.472	0.184
		*p*	0.676	0.908	0.703	0.907
		*df*	3	3	3	3
	CO_2_ × replacement	*F*	0.491	0.597	0.464	1.36
		*p*	0.69	0.62	0.709	0.267
		*df*	3	3	3	3
	CO_2_ × N × replacement	*F*	0.033	0.374	0.027	0.736
		*p*	0.992	0.772	0.994	0.536
		*df*	3	3	3	3

Df represents degrees of freedom, * represents *p* < 0.05, ** represents *p* < 0.01

**Table 3:** Results of three-way ANOVA for the seed characters of the alien invasive plants common ragweed according to CO_2_, N, and replacement levels: seed yield, seed mean weight.

Factors		Seed Number	Seed Total Weight	Seed Mean Weight
CO_2_	*F*	0.017	0.344	0.181
	*p*	0.896	0.560	0.673
	*df*	1	1	1
N	*F*	14.821	1.241	12.024
	*p*	<0.001 **	0.271	0.001 **
	*df*	1	1	1
replacement	*F*	2.316	2.135	0.330
	*p*	0.088	0.109	0.804
	*df*	3	3	3
CO_2_ × N	*F*	1.654	0.999	0.092
	*p*	0.205	0.323	0.763
	*df*	1	1	1
N × replacement	*F*	0.437	0.926	0.689
	*p*	0.727	0.436	0.563
	*df*	3	3	3
CO_2_ × replacement	*F*	0.806	0.360	0.083
	*p*	0.497	0.782	0.969
	*df*	3	3	3
CO_2_ × N × replacement	*F*	1.203	0.977	1.885
	*p*	0.319	0.412	0.145
	*df*	3	3	3

Df represents degrees of freedom, * represents *p* < 0.05, ** represents *p* < 0.01

## Data Availability

The data presented in this study are available on request from the corresponding author.
